# Medical Examining Board in Georgia

**Published:** 1898-09

**Authors:** 


### MEDICAL EXAMINING BOARD IN GEORGIA.

Georgia now has three Boards of Medical Examiners, representing the three different schools of medicine, viz.: Regular, Homeopathic, and Eclectic.

At the last meeting of the Regular Board of Examiners, representing the regular school, a resolution was passed making THE ATLANTA MEDICAL AND SURGICAL JOURNAL the official organ of the Board, and in which the regular proceedings will be published. The Secretary, Dr. E. R. Anthony, of Griffin, Ga., will in the future furnish data concerning the meetings of the Board, which will thus be a guide to medical men all over the State. At the last meeting, held in Atlanta on July 6, a large number of physicians came before the Board, seeking the necessary qualifications for practicing in the State. We are sorry not to be able to publish a list of those who were given certificates. The following is the examination that was given :

MATERIA MEDICA AND THERAPEUTICS—BY DR. W. A. O'DANIEL.

1. How is quinine an antiperiodic, and how is it chiefly eliminated ?

2. What are the official preparations of hydrastis, the dose of each, and its therapeutics ?

3. What crystallizable principle is contained in pipsissewa?

4. Give antidotes for poisoning by phosphorus ?

5. Name the most useful emetics, and the dose and mode of administration of each.

6. Give the physiological action and therapeutics of digitalis.

7. Name the drugs which tend to be cumulative in the system during long periods of administration.

CHEMISTRY.—DR. E. R. ANTHONY.

1. Give Avogadro's law.

2. Name several electro-positive elements and several electro-negative elements.

3. Combine H and a negative element or radical and what is produced ?

4. What is hydroxyl? Give me its formula.

5. What kind of albumin is found in the urine in albuminuria? Give a good test for same.

PHYSIOLOGY.—DR. E. R. ANTHONY.

1. What is the origin of the colorless blood corpuscles? From what are the colored corpuscles derived ?

2. What is the size and weight of the heart? What is the capacity of its chambers? What is the comparative thickness of the walls of the right and left ventricles?

3. What is the object of respiration? Where does this exchange of oxygen and carbon dioxid take place?

4. Briefly describe the digestion of albumin.

5. Select a cranial nerve and give me its function.

### ANATOMY.—DR. F. M. RIDLEY.

1. Name movable joints and give kinds of movements admitted in same.

2. Name muscles composing quadriceps extensor femoris, and give origin and insertion of each.

3. Describe relation of external carotid and name its branches.

4. Name nerves composing brachial plexus and give distribution.

5. Describe solar plexus. Give origin, course, and distribution of pnemogastric nerve.

### SURGERY.—DR. F. M. RIDLEY.

1. Define inflammation, and describe changes occurring in surrounding tissues and blood-vessels during same.

2. Describe sapremia, septicemia, pyemia. Give distinguishing symptoms.

3. Name varieties of fractures. Differential diagnosis between fracture and dislocation. Name cardinal principles in treatment of fracture. Give treatment of a recent compound fracture of leg.

4. Give pathology, diagnosis and treatment, also differential diagnosis, of chancre and chancroid.

5. What is hernia? Give differential diagnosis between inguinal hernia and hydrocele. Name coverings from without inward of an oblique inguinal hernia. Describe an operation for the radical cure.

GYNECOLOGY.—DR. J. B. S. HOLMES.

1. Describe the gross anatomy of the uterus.

2. Describe the blood supply of the uterus.

3. Describe the round ligaments.

4. What is pudendal hernia? and what is the treatment?

5. Describe briefly the operation of vaginal hysterectomy.

OBSTETRICS—DR. J. B. S. HOLMES.

1. What are the causes of nausea and vomiting of pregnancy? What is the treatment?

2. How should the hand of an obstetrician be prepared before examining a woman?

3. When may an anesthetic be used in normal labor? What anesthetic is to be preferred?

4. What should a physician carry with him in attending an obstetric case?

5. After a delivery when should a physician leave and when should he return?

PRACTICE.—DR. A. A. SMITH.

1. Give symptoms and appropriate treatment for chorea?

2. What abnormal constituents would you expect to find in the examination of the urine in diabetes?

3. Give the most prominent diagnostic symptoms in yellow fever.

4. What is pericarditis? Give symptoms and treatment for same.

5. Give definition of hematuria.

6. Give symptoms and treatment of acute peritonitis.

7. Give symptoms and treatment of cholera infantum.

8. Give appropriate treatment for typhoid fever.

---

IN our last issue an error occurred which should have been corrected. In our editorial on "Consolidation of Medical Colleges" it should have read, "the Niagara University Medical College and the Buffalo University Medical School have consolidated under the name of the Medical Department of the University of Buffalo."

